# Integrating Real-World Evidence, Patient-Reported Outcomes and Health Economics into Evidence-Based Public Health—A Narrative Review and Conceptual Framework

**DOI:** 10.3390/clinpract16100190

**Published:** 2026-10-13

**Authors:** Mariola Borowska, Piotr Bromber, Tomasz Kopiec, Magdalena Malinowska-Stelmachowska, Wojciech Miazga, Teresa Pakulska, Sylwia Jędrzejczyk-Kowalska, Urszula Religioni

**Affiliations:** 1 School of Public Health, Center of Postgraduate Medical Education, 01-813 Warsaw, Poland; piotr.bromber@cmkp.edu.pl (P.B.); tomasz.kopiec@cmkp.edu.pl (T.K.); magdalena.stelmachowska@szpital-orlowskiego.pl (M.M.-S.); wojciech.miazga@cmkp.edu.pl (W.M.); sylwia.kowalska@uckwum.pl (S.J.-K.); urszula.religioni@cmkp.edu.pl (U.R.); 2 Centre of Postgraduate Medical Education, Prof. W. Orłowski Memorial Hospital, 00-416 Warsaw, Poland; 3 Department of Competition Strategy, Collegium of Business Administration, Warsaw School of Economics, 02-554 Warsaw, Poland; tpakuls@sgh.waw.pl; 4 Central Clinical Hospital, Medical University of Warsaw, 01-813 Warsaw, Poland

**Keywords:** evidence-based public health, real-world evidence, real-world data, patient-reported outcome measures, patient-reported experience measures, health technology assessment, value-based healthcare, learning health systems, health economics

## Abstract

Contemporary healthcare systems operate in an environment characterized by the rapid development of digital technologies and the growing availability of health data. While these developments may increase the complexity of health policy decision-making, they also create new opportunities to support this process. In addition to clinical trial findings, RWD/RWE, PROMs, and PREMs, as well as analyses in the fields of health economics, HTA, and VBHC, are becoming increasingly important. The aim of this article is to present the current state of knowledge on the integration of these data and evidence sources within the EBPH approach and to discuss their relevance to the planning, implementation, and evaluation of health interventions. Based on a review of the literature, we propose an original conceptual model for health policy decision-making—the Integrated Evidence-Based Public Health Framework, which integrates epidemiological data, RWE, patient-reported information, and economic analyses within a single decision-support framework. The proposed model is consistent with the LHS concept and may provide a useful tool for supporting rational health policy planning and assessing the value of health interventions.

## 1. Introduction

Contemporary healthcare systems operate in the context of demographic, epidemiological, and technological changes that increasingly affect decision-making at clinical, organizational, and system levels. Population ageing, the growing burden of chronic disease and multimorbidity, rising patient expectations, and persistent financial and workforce constraints increase the need for decisions that maximize health benefits while ensuring efficient use of available resources. The Learning Health Systems (LHS) concept responds to these challenges by promoting the continuous use of routinely generated data and scientific evidence to improve clinical practice, healthcare organization, and health policy [1,2].

The rapid development of digital technologies has also created an extensive health data ecosystem. Electronic health records, disease registries, administrative and reimbursement databases, wearable devices, telemedicine solutions, and mobile applications generate large amounts of information on population health and healthcare delivery. Patient-reported information, including Patient-Reported Outcome Measures (PROMs) and Patient-Reported Experience Measures (PREMs), complements these data by capturing outcomes and experiences that are directly relevant to patients [1,3].

Despite the growing availability of data, healthcare decision-making often remains fragmented. Randomized clinical trials remain a fundamental source of evidence, but their findings do not always reflect outcomes achieved in routine practice. Real-World Data (RWD) and Real-World Evidence (RWE) can complement trial evidence by providing information on effectiveness, safety, and healthcare utilization under real-world conditions [1]. Together with epidemiological data, PROMs/PREMs, and economic analyses, these sources provide a broader basis for public health decision-making than any single source considered in isolation.

The evaluation of health interventions is therefore increasingly multidimensional. Health economics, Health Technology Assessment (HTA), and Value-Based Healthcare (VBHC) extend assessment beyond clinical effectiveness by incorporating resource use, costs, organizational implications, quality of life, and outcomes that matter to patients and society [1,4].

However, Evidence-Based Public Health (EBPH), LHS, RWE, PROMs/PREMs, HTA, health economics, and VBHC are still often applied within separate analytical or decision-making processes. These approaches address complementary dimensions of evidence-informed healthcare: LHS emphasizes continuous learning from routinely generated data, HTA provides structured multidisciplinary assessment, and VBHC focuses on outcomes that matter to patients in relation to resources used. Yet, they do not by themselves provide a unified public health decision-making structure that explicitly links population needs, real-world evidence, patient-reported information, economic and broader HTA considerations, implementation decisions, and post-implementation feedback within a single iterative process.

This represents the conceptual gap addressed in the present article. We therefore aim to synthesize current knowledge on the integration of RWE, patient-reported outcomes and experiences, health economics, and population-level data in public health decision-making. Based on this synthesis, we propose the Integrated Evidence-Based Public Health (IEBPH) Framework, a conceptual model that combines these evidence domains within a continuous process of identifying population health needs, informing policy and implementation decisions, evaluating their consequences, and feeding newly generated evidence back into subsequent decision-making.

### 1.1. Review Method

This study was conducted as a narrative review aimed at synthesizing current knowledge on the integration of real-world data and evidence (RWD/RWE), patient-reported outcomes and experiences (PROMs/PREMs), health economics, health technology assessment (HTA), value-based healthcare (VBHC), learning health systems (LHS), and evidence-based public health (EBPH).

The literature search was conducted between May and July 2026 using PubMed/MEDLINE, Scopus, and Web of Science. Additional searches of relevant institutional and regulatory sources, including the U.S. Food and Drug Administration (FDA), European Medicines Agency (EMA), Health Technology Assessment international (HTAi), and European Commission websites, were performed to identify methodological, regulatory, and policy documents relevant to the topics covered in the review. The searches were conducted across the principal thematic domains addressed in the review in order to identify publications relevant not only to the individual evidence sources but also to their integration within healthcare and public health decision-making.

The search strategy used combinations of terms related to “evidence-based public health,” “real-world data,” “real-world evidence,” “patient-reported outcomes,” “patient-reported outcome measures,” “patient-reported experience measures,” “health economics,” “health technology assessment,” “value-based healthcare,” “learning health systems,” “health data,” and “public health decision-making.” Boolean operators were used to combine concepts where appropriate.

No strict publication-date restriction was applied, as the review aimed to include both recent evidence and seminal publications relevant to the development and definition of the principal concepts discussed. Priority was given to recent peer-reviewed original studies, systematic and scoping reviews, methodological papers, and authoritative institutional or regulatory documents directly relevant to the integration and use of different evidence sources in healthcare and public health decision-making. Older publications were retained when they represented seminal contributions to key concepts or methods. No formal restriction based on study design was applied, given the conceptual and integrative nature of the review.

Publications were selected on the basis of their conceptual and practical relevance to the objectives of the review. In particular, preference was given to publications addressing the role, integration, interpretation, or application of RWD/RWE, PROMs/PREMs, health economics, HTA, VBHC, LHS, and EBPH in healthcare-system or population-level decision-making. Studies focusing exclusively on narrowly defined clinical questions without a clear relationship to evidence integration, health-system evaluation, or public health decision-making were not prioritized. Titles, abstracts, and full texts were considered as appropriate to determine whether publications contributed substantively to one or more of the thematic domains covered by the review. Where relevant, reference lists of key reviews, methodological papers, and institutional documents were also examined to identify additional seminal or highly relevant publications. The selected literature was synthesized thematically to identify the principal evidence domains, their complementary roles, and the relationships relevant to public health decision-making. Particular attention was paid to areas of conceptual overlap and complementarity between EBPH, LHS, RWE, PROMs/PREMs, health economics, HTA, and VBHC, as well as gaps in existing approaches to integrating these domains within a single public health decision-making process. This synthesis subsequently informed the development of the proposed Integrated Evidence-Based Public Health Framework.

### 1.2. The Health Data Ecosystem—From Traditional Sources of Information to Digital Health

Public health decision-making has historically relied on the analysis of data on population health. Traditionally, the primary sources of information included demographic statistics, mortality registries, infectious disease surveillance data, and epidemiological studies, which enabled the identification of major health problems, the monitoring of morbidity and mortality trends, and the planning of preventive interventions. However, advances in information technology have fundamentally changed the way health information is collected, processed, and used, leading to the emergence of a complex health data ecosystem [5].

The contemporary health data ecosystem encompasses information derived from multiple complementary sources, including Electronic Health Records (EHRs), disease registries, administrative and reimbursement databases, population health monitoring systems, data generated by wearable devices, mobile applications, and telemedicine solutions. Integrating these data makes it possible to analyze the course of care and treatment and the use of healthcare resources, the quality of care provided, and the long-term outcomes of health interventions. Increasingly, these data are being used both in scientific research and in the planning and management of healthcare systems [6].

An important component of the contemporary health data ecosystem is information reported directly by patients. PROMs and PREMs provide information that is not available from conventional medical records. They make it possible to assess the impact of disease and treatment on patients’ daily functioning, quality of life, psychological well-being, and satisfaction with healthcare. Incorporating these data into routine health information systems is needed to improve quality of care and facilitate better alignment of interventions with patients’ needs.

Digital transformation has also changed the role of health data, which are no longer used solely for reporting purposes. Advances in Big Data analytics, artificial intelligence, and machine learning have made it possible to identify patterns of disease occurrence, forecast demand for healthcare services, monitor quality of care, and support clinical and organizational decision-making. Therefore, it is increasingly emphasized that the value of health data lies primarily in the ability to integrate them and transform them into actionable information that supports decision-making [5,7].

Despite substantial technological progress, numerous barriers continue to hinder the full realization of the potential of health data. The most important include limited interoperability between information systems, variability in data quality, the lack of standardized approaches to coding medical information, privacy-related concerns, and limited capacity to link data from different sources. In many countries, clinical, epidemiological, administrative, and economic information continues to be stored and managed in separate systems, making comprehensive analyses difficult and limiting the use of data to support health policy and healthcare system management [6,7].

These challenges are particularly relevant to the development of Learning Health Systems (LHS), in which data generated during routine care are continuously analyzed and reused to support improvement in clinical practice, healthcare organization, and health policy. Effective LHS implementation depends on appropriate digital infrastructure, governance, organizational capacity, stakeholder engagement, and high-quality interoperable data [1,8].

Access to data alone, however, does not constitute evidence. Data must be analyzed using appropriate methodological approaches before they can inform reliable conclusions and decisions. This distinction is central to the concepts of Real-World Data (RWD) and Real-World Evidence (RWE), which are discussed in the following section.

### 1.3. Real-World Data and Real-World Evidence: From Data to Reliable Scientific Evidence

The development of digital health information systems has created opportunities to collect vast amounts of data generated during routine patient care. These data, referred to as RWD, include information derived from electronic health records, disease registries, administrative and reimbursement databases, insurance data, pharmacovigilance systems, wearable devices, and patient-reported data. RWD therefore refers to data collected in routine healthcare settings, whereas RWE refers to evidence generated through the appropriate analysis and interpretation of RWD. More broadly, data represent collected information, while evidence refers to findings derived from the systematic analysis and interpretation of that information. Appropriately designed analyses of RWD can generate RWE, providing scientific evidence on the effectiveness, safety, and utilization of health interventions under real-world healthcare conditions [9,10].

The importance of RWE has steadily increased as the limitations of randomized controlled trials (RCTs) have become more widely recognized. Although RCTs remain a fundamental source of evidence on the efficacy of medical interventions because of their high internal validity, their findings do not always reflect the outcomes achieved in routine clinical practice. Strict eligibility criteria, controlled study conditions, and relatively short follow-up periods may limit the generalizability of findings to real-world patient populations, including older adults, patients with multimorbidity, those receiving multiple concomitant therapies, and individuals with varying levels of adherence to treatment recommendations [9]. In this context, RWE should be regarded as a complement rather than an alternative to randomized trials, enabling the assessment of the real-world effectiveness of health interventions rather than solely their efficacy under experimental conditions [11].

In recent years, there has been a marked shift in the approach of regulatory authorities towards the use of RWE. Both the European Medicines Agency (EMA) and the U.S. Food and Drug Administration (FDA) have developed guidance on the use of real-world data in regulatory processes, emphasizing the need to ensure adequate data quality, transparency of analytical methods, and appropriate measures to minimize the risk of bias. RWE is now used in pharmacovigilance, the assessment of the effectiveness of health technologies, clinical trial planning, analyses of healthcare utilization, and support for reimbursement decisions and HTA [9,10].

The growing importance of RWE is also evident in the field of public health [12]. Real-world data can be used to evaluate the effectiveness of preventive programs, analyze health inequalities, monitor quality of care, and identify populations at particularly high risk of adverse health outcomes. Unlike conventional clinical trials, real-world studies can provide insights into the functioning of entire healthcare systems while accounting for the influence of organizational, social, and economic factors on health outcomes. RWE is therefore becoming a key source of information for supporting population-level decision-making.

Despite its numerous advantages, the use of RWE involves important methodological challenges. Data derived from routine clinical practice are often incomplete and heterogeneous and are collected primarily for administrative or clinical rather than research purposes. The absence of randomization increases the risk of selection bias, confounding, and measurement error. The reliability of findings depends on data quality, appropriate study design, and the application of suitable statistical methods. Current guidelines emphasize that assessment of data quality and fitness for purpose should be an integral component of every study using RWD [12,13].

Within the proposed integrated approach, RWE contributes a distinct form of evidence that helps assess whether effects observed under controlled conditions are reproduced in routine practice and how these effects vary across populations and healthcare settings. It does not, however, capture all dimensions relevant to decision-making. In particular, clinical and administrative data may provide limited information on outcomes and experiences that are directly meaningful to patients. These dimensions are addressed by PROMs and PREMs.

### 1.4. Patient-Reported Outcomes (PROMs and PREMs) as a Component of Health Intervention Value Assessment

For many years, the effectiveness of health interventions was assessed primarily using clinical indicators such as mortality, morbidity, complication rates, and laboratory test results. Although these measures remain fundamental to the assessment of treatment outcomes, they do not fully capture the impact of disease and health interventions on patients’ daily functioning. Particularly in chronic, oncological, neurological, and rheumatological conditions, quality of life, functional status, psychological well-being, and the ability to perform everyday activities are becoming as important as prolonging survival. Consequently, the past two decades have seen rapid development of tools designed to systematically incorporate the patient perspective into the assessment of healthcare outcomes [14,15].

PROMs and PREMs are among the most important tools used for this purpose. PROMs capture information reported directly by patients on health-related quality of life, symptom severity, physical, psychological, and social functioning, and the effects of treatment, without interpretation by healthcare professionals. PREMs, in turn, focus on patients’ experiences with the organization and delivery of healthcare, including communication with healthcare professionals, access to services, coordination of care, and satisfaction with care. The two measures therefore address different but complementary dimensions: PROMs describe outcomes as experienced by patients, whereas PREMs describe patients’ experiences of the care process itself [15,16].

The growing importance of PROMs is reflected in both research and clinical practice. They are increasingly used as primary or secondary endpoints in clinical trials, as measures for monitoring quality of care, and as tools supporting therapeutic decision-making. In many countries, patient-reported data are systematically collected in national clinical registries and used to compare treatment outcomes across healthcare providers and to evaluate the effectiveness of implemented models of care. Systematic reviews indicate that the routine use of PROMs improves communication between patients and healthcare professionals, facilitates the identification of unmet health needs, and may contribute to improved treatment outcomes and quality of care [17].

The relevance of patient-reported data extends beyond individual clinical care. These data are increasingly used to assess healthcare system performance and in HTA processes. Their contribution is particularly important when conventional clinical measures do not fully reflect differences in functioning, quality of life, treatment burden, or experience of care. In this way, PROMs and PREMs complement clinical and real-world evidence by adding information on whether health interventions produce outcomes that are meaningful from the patient perspective [18]. This role is also consistent with the principles of VBHC, in which outcomes that matter to patients constitute a central component of value assessment.

Despite their benefits, the implementation of PROMs and PREMs in routine practice continues to face numerous barriers. The most frequently reported challenges include lack of standardization of measurement instruments, difficulties in integrating patient-reported data with electronic health records, limited interoperability of information systems, and the absence of standardized methods for interpreting results. Additional challenges include response burden, incomplete data, differences in instrument selection, and the need to ensure that collected information is sufficiently reliable and actionable for clinical, organizational, and policy purposes. Literature reviews emphasize that successful implementation of PROMs cannot be achieved through technological solutions alone. Organizational changes, engagement of healthcare professionals, and active patient participation in the assessment of quality of care are also required [17,18].

In the integrated evidence-based approach, PROMs and PREMs therefore serve a distinct function: they introduce the patient perspective into the assessment of health outcomes and care delivery. However, patient-reported outcomes alone do not determine whether an intervention represents an efficient, affordable, or feasible use of healthcare resources. These dimensions are addressed through health economics, HTA, and VBHC, discussed in the following section.

### 1.5. Health Economics, HTA, and VBHC

Growing population health needs and limited financial resources increasingly require contemporary healthcare systems to select interventions that provide the greatest health benefits while ensuring the rational use of available resources. Decision-making cannot be based solely on the assessment of clinical effectiveness but should also take into account economic, organizational, and social considerations, as well as the impact of interventions on patients’ quality of life. This approach underpins health economics, which aims to support the efficient allocation of resources by evaluating the costs and outcomes of different diagnostic, therapeutic, and preventive strategies [19].

Health Technology Assessment provides a broader framework for informing such decisions. According to the international definition, HTA is a multidisciplinary process that systematically assesses the value of health technologies across clinical, economic, organizational, social, ethical, and legal dimensions [20]. Its scope therefore extends beyond economic evaluation and may draw on evidence from randomized trials, RWE, epidemiological studies, patient-reported information, economic analyses, and other sources relevant to the decision context. The purpose of HTA is to support transparent decisions concerning the introduction, reimbursement, implementation, use, or disinvestment of health technologies.

Economic evaluation constitutes one component of this broader assessment. Common approaches include cost-effectiveness analysis, cost–utility analysis, and cost-benefit analysis. Cost–utility analyses frequently use quality-adjusted life years, which combine length of life with health-related quality of life and enable comparisons across interventions. Although QALYs provide a standardized measure of health benefit, they do not capture every dimension that may be relevant to patients or healthcare systems, including experiences of care, treatment burden, distributional considerations, and organizational or social consequences [21].

VBHC approaches the question of value from a different perspective. Rather than focusing primarily on the assessment of a technology at a particular decision point, VBHC emphasizes the outcomes achieved for patients in relation to the resources used across the care pathway. This shifts attention from the volume of services delivered toward outcomes that matter to patients and the costs associated with achieving them. Operationalizing VBHC consequently requires systematic measurement of clinical outcomes, PROMs, PREMs, and resource use [22].

Although health economics, HTA, and VBHC are closely related, they address different aspects of healthcare decision-making. Health economics offers analytical methods for comparing costs and consequences; HTA places these analyses within a broader multidisciplinary assessment, whereas VBHC focuses on the value generated for patients across care delivery. Keeping these distinctions clear is important when different forms of evidence are brought together for policy decisions.

Practical implementation remains challenging. Common barriers include limited interoperability of information systems, lack of standardized outcome measures, difficulties in linking clinical, patient-reported, and economic data, and fragmentation of analytical and decision-making processes [23,24]. As a result, evidence relevant to the same policy question may be produced in separate clinical, economic, organizational, and patient-centered streams and considered at different stages or by different institutions. This problem provides the immediate rationale for the integrated public health decision-making framework.

### 1.6. An Integrated Public Health Decision-Making Model: A Proposed Conceptual Framework

The areas discussed in the preceding sections, including EBPH, RWE, patient-reported data, health economics, HTA, and VBHC, have developed with different aims and methods. In practice, they are often used separately, even though public health decisions usually require consideration of several types of evidence at the same time. Clinical, epidemiological, economic, organizational, and patient-reported information may be collected and assessed by different institutions, which can make it difficult to form a complete picture of the value and effects of health interventions.

Existing approaches address important parts of this process, but from different perspectives. EBPH focuses on the use of the best available evidence in population-level decision-making. LHS emphasizes continuous learning from data generated in routine care. HTA provides a multidisciplinary assessment of health technologies, while VBHC focuses on outcomes that matter to patients in relation to the resources used. The proposed framework brings these perspectives together in one public health decision process.

The originality of the proposed IEBPH Framework does not lie in introducing new categories of evidence or replacing existing approaches such as EBPH, LHS, HTA, or VBHC. Rather, its conceptual contribution lies in integrating these complementary perspectives within a single, explicitly structured, and iterative public health decision-making process. In contrast to approaches that primarily focus on continuous learning from routinely generated data, multidisciplinary assessment of individual health technologies, or patient-centered value, the IEBPH Framework begins with the identification of population health needs and explicitly links these needs with epidemiological evidence, RWD/RWE, patient-reported information, economic evaluation and HTA, implementation decisions, and post-implementation feedback. The framework therefore serves as an overarching integrative structure that connects evidence generation, appraisal, implementation, and reassessment at the population and health-system levels.

**Figure 1:**
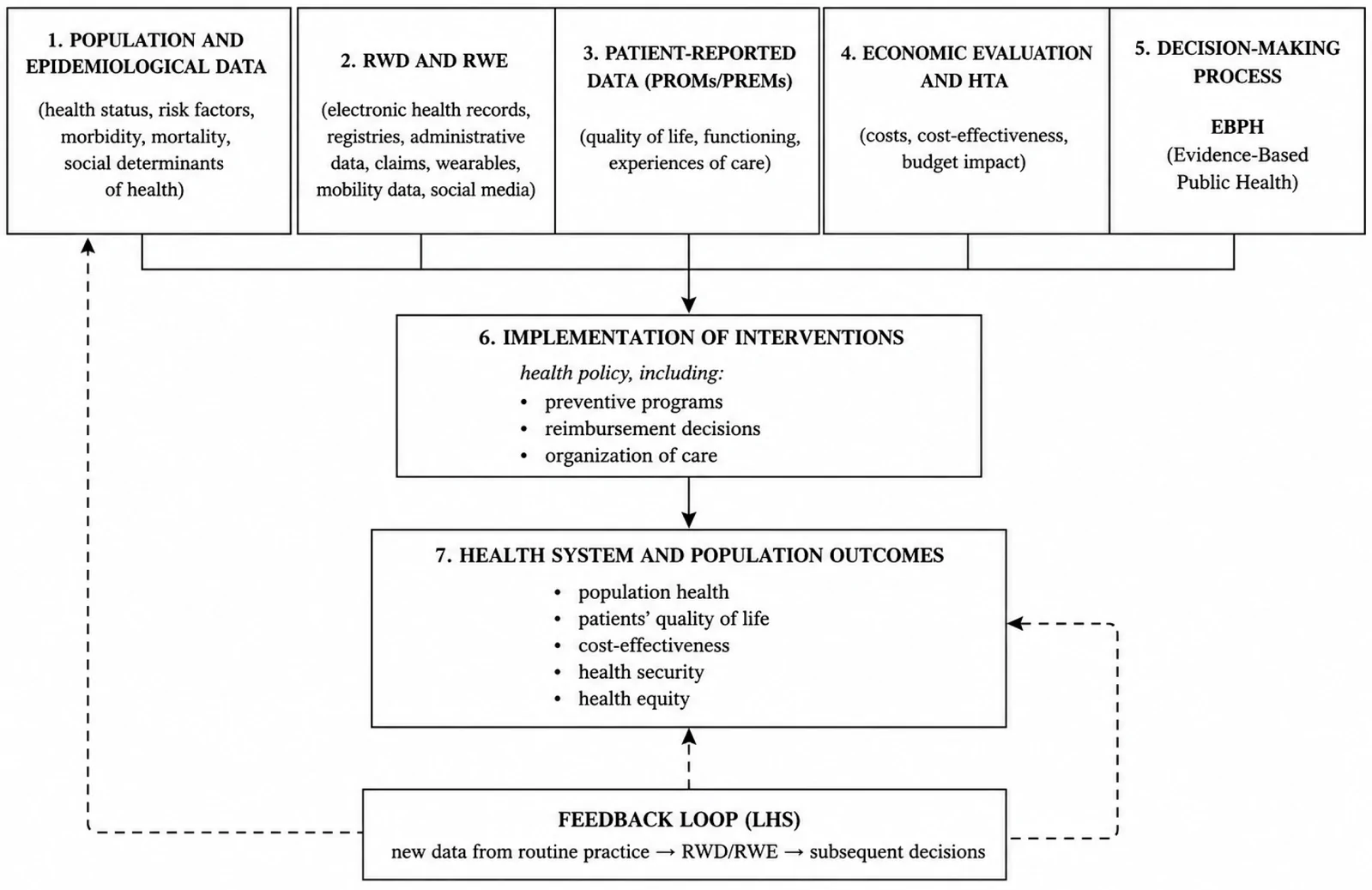
Integrated Evidence-Based Public Health Framework (IEBPH Framework).

Based on the literature review, we propose the Integrated Evidence-Based Public Health Framework (IEBPH Framework), a conceptual model that integrates complementary sources of data and scientific evidence into the health policy decision-making process (Figure 1).

The model is based on five interconnected pillars. The first four represent complementary sources of data and evidence, while the fifth integrates these inputs into the public health decision-making process. Together, these pillars form a continuous framework for planning, implementing, and evaluating health interventions.

1. Population and epidemiological data

The first pillar comprises information on population health status, risk factors, morbidity, mortality, and the social determinants of health. Their main role is to identify health problems, define priority populations, and determine where intervention may be needed.

2. RWD and RWE

RWD include data generated during routine healthcare delivery, such as electronic health records, clinical registries, reimbursement data, and administrative databases. Their analysis may generate RWE on the effectiveness, safety, and use of interventions in routine practice. This evidence helps determine whether effects observed in controlled studies are also seen in real-world populations and healthcare settings.

3. Patient-reported data (PROMs/PREMs)

PROMs and PREMs provide information on patients’ quality of life, functioning, symptoms, and experiences of care. They add the patient perspective to clinical and administrative evidence and help assess whether an intervention produces outcomes that are meaningful to those receiving care.

4. Economic evaluation and HTA

Economic evaluation and HTA provide information on costs, cost-effectiveness, budget impact, and broader clinical, organizational, ethical, legal, and social consequences. It helps determine whether an intervention is effective, affordable, feasible, and appropriate in a given healthcare system.

5. Decision-making process

The final component of the model is an integrated decision-making process in which the findings of epidemiological, clinical, and economic analyses, together with patient-reported data, are synthesized and interpreted in the context of the legal, organizational, and strategic conditions of the healthcare system. Population and epidemiological data help define the health problem and target population, RWE informs the performance of interventions in routine practice, patient-reported data provide the patient perspective, and economic evaluation and HTA support the assessment of costs, feasibility, and broader consequences. This integrated assessment can support health policy planning, reimbursement decisions, the design and implementation of preventive programs, and the monitoring of healthcare quality and efficiency.

An important component of the proposed model is a feedback loop. The outcomes of implemented interventions generate new real-world data, which can be used to evaluate their effectiveness, safety, resource use, and patient-reported outcomes in routine practice. These findings may then inform the continuation, modification, expansion, or discontinuation of an intervention. This mechanism is consistent with the principles of LHS and allows decision-making to be understood as a continuous learning cycle rather than as a series of one-off activities.

The proposed conceptual framework is intended to be broadly applicable and may be relevant to national health policy, regional healthcare planning, hospital management, and the implementation of preventive programs. It may also provide a common structure for linking evidence used in HTA, quality-of-care assessment, and value-based healthcare with broader public health decision-making. However, its feasibility and applicability require further empirical evaluation across different healthcare systems and decision-making contexts.

It should be emphasized, however, that the proposed model is conceptual in nature and requires further empirical validation. Future research should assess its feasibility and applicability across different healthcare systems and determine the extent to which the integration of multiple data sources influences the quality of healthcare decision-making, the efficiency of resource utilization, and health outcomes.

### 1.7. Challenges and Prospects for Implementing the Integrated Decision-Making Model

The growing availability of health data and the rapid development of analytical methods create new opportunities to support decision-making in healthcare. At the same time, combining epidemiological, clinical, economic, and patient-reported information remains an important organizational, technological, and methodological challenge. Despite numerous healthcare digitalization initiatives, many systems continue to rely on fragmented data sources that do not provide full interoperability or enable comprehensive analysis of available information [25,26].

One of the fundamental limitations remains data quality. Data generated in routine clinical practice are collected primarily for diagnostic and administrative, rather than research, purposes. Consequently, common problems include incomplete information, differences in data coding practices, documentation errors, and a lack of standardization across healthcare providers. These limitations may affect the reliability of RWE analyses and hinder comparisons of outcomes across regions and healthcare systems [27].

A related challenge concerns differences in the quality and certainty of evidence obtained from different sources. RCTs, observational studies, RWE analyses, patient-reported data, and economic evaluations may differ in methodological quality, risk of bias, relevance to the target population, and degree of uncertainty. Their findings may also be inconsistent. In such situations, evidence should not be treated as equally reliable. Differences in study design, populations, data sources, outcome definitions, and analytical methods should be considered when interpreting conflicting results. Where uncertainty remains substantial, additional data collection or further evaluation may be needed before a final decision is made.

Another major challenge is the interoperability of health information systems. Effective use of data requires the ability to securely link information from electronic health records, clinical registries, reimbursement databases, public health surveillance systems, and patient-reported data. The lack of common data exchange standards substantially limits the ability to conduct comprehensive analyses supporting decision-making. In response to these challenges, the European Union is developing the European Health Data Space (EHDS) [28], which aims to establish a secure legal and technical framework for the secondary use of health data for research, planning, and healthcare system management.

Privacy and ethical considerations also remain important. The integration of large datasets requires appropriate levels of information security, compliance with applicable data protection regulations, and transparent rules governing access to data. Building trust among patients and the public regarding the use of health data is one of the fundamental prerequisites for the development of modern health information systems [29].

AI and machine learning methods have also become increasingly important in recent years. These tools enable the analysis of very large datasets, the identification of complex relationships, and the prediction of future population health needs. They can support the identification of high-risk groups, evaluation of health program effectiveness, optimization of resource utilization, and personalization of interventions. Experts emphasize that AI models should support rather than replace clinical judgment or decisions made by public health professionals. Model transparency, interpretability of results, and independent validation of algorithms before their implementation in practice are therefore of critical importance [30].

The future of public health is likely to increasingly reflect the LHS model, in which data generated during routine care are continuously transformed into scientific evidence and used to improve the organization of healthcare services and health policy. Such an approach supports repeated evaluation and adjustment of decisions as new evidence becomes available [2,6,31].

The proposed IEBPH Framework is consistent with this direction, but its practical implementation will depend on more than access to data. It will require adequate digital infrastructure, data standardization, appropriate analytical competencies, transparent methods for assessing evidence quality and uncertainty, and collaboration among decision-makers, clinicians, epidemiologists, health economists, and patients. Its feasibility and performance should be evaluated empirically across different healthcare systems and decision-making contexts.

## 2. Conclusions

Contemporary public health decision-making requires the integration of multiple complementary sources of evidence, including clinical trial findings, RWD/RWE, PROMs/PREMs, and economic analyses. Their combined use within an EBPH approach may support a more comprehensive assessment of health interventions and better-informed planning and resource allocation.

In response to the identified conceptual gap, we propose the Integrated Evidence-Based Public Health Framework, which integrates epidemiological data, RWE, patient-reported information, and economic and HTA evidence within a single decision-making structure. A feedback mechanism informed by the LHS concept enables newly generated data to contribute to the reassessment of implemented interventions and subsequent decisions.

The IEBPH Framework should currently be regarded as a conceptual model whose feasibility, applicability, acceptability, and performance require empirical evaluation across different healthcare systems and policy contexts. Such evaluation should determine whether the framework improves the integration and interpretation of evidence and contributes to better decision-making, resource use, and health outcomes.

Its practical application should also account for legal, organizational, strategic, societal, and patient-related considerations. Widespread adoption should therefore not be recommended until the framework’s feasibility and added value have been demonstrated empirically.

## Data Availability

No new data were created or analyzed in this study. Data sharing is not applicable to this article.
